# Healthcare Professionals' Responses to Complaints: A Qualitative Interview Study With Patients, Carers and Healthcare Professionals Using the Theoretical Domains Framework and COM‐B Model

**DOI:** 10.1111/hex.70118

**Published:** 2024-12-08

**Authors:** Vivi Antonopoulou, Paulina M. Schenk, Alison R. McKinlay, Paul Chadwick, Carly Meyer, Beckie Gibson, Falko F. Sniehotta, Fabiana Lorencatto, Ivo Vlaev, Angel M. Chater

**Affiliations:** ^1^ NIHR Policy Research Unit in Behavioural and Social Sciences, Department of Clinical, Education and Health Psychology, Centre for Behaviour Change University College London London UK; ^2^ NIHR Policy Research Unit in Behavioural and Social Sciences, Population Health Sciences Institute, Faculty of Medical Sciences Newcastle University Newcastle upon Tyne UK; ^3^ Public Health, Social and Preventive Medicine, Centre of Preventive Medicine and Digital Health, Medical Faculty Mannheim Heidelberg University Mannheim Germany; ^4^ NIHR Policy Research Unit in Behavioural and Social Sciences, Behavioural Science Group, Warwick Business School University of Warwick Coventry UK; ^5^ NIHR Policy Research Unit in Behavioural and Social Sciences, Centre for Health, Wellbeing and Behaviour Change University of Bedfordshire Bedford UK

**Keywords:** behavioral research, COM‐B, health services, patient feedback, patient safety, TDF

## Abstract

**Background:**

Patient complaints in healthcare settings can provide feedback for monitoring and improving healthcare services. Behavioural responses to complaints (e.g., talking or apologising to a patient) can influence the trajectory of a complaint for instance, whether a complaint is escalated or not. We aimed to explore healthcare professional (HCP) and service user (patient and carer) views on complaints' management and the perceived factors influencing responses to complaints within a healthcare setting by applying behavioural frameworks.

**Method:**

A qualitative study was conducted using online or phone‐based interviews with eleven HCPs and seven patients or carers. All participants (*N* = 18) had experience responding to or submitting a formal complaint in secondary and tertiary public healthcare settings in the United Kingdom. The interviews were structured using the Capability‐Opportunity‐Motivation‐Behaviour (COM‐B) Model. We analysed the transcripts using inductive thematic analysis. Then, themes were deductively mapped onto the COM‐B Model and the more granular Theoretical Domains Framework (TDF).

**Results:**

Ten themes were generated from the analysis representing the influences on HCPs' responses to complaints from HCP and patient/carer perspectives. This included (with TDF/COM‐B in brackets): ‘Knowledge of complaint procedure’ (Knowledge/Capability), ‘Training and level of skill in complaints handling’ (Skills/Capability), ‘Regulation of emotions associated with complaints’ (Behavioural regulation/Capability), ‘Confidence in handling complaints’ (Beliefs about capabilities/Motivation), ‘Beliefs about the value of complaints’ (Beliefs about consequences/Motivation) and ‘Organisational culture regarding complaints’ (Social influences/Opportunity). Staff highlighted strong support systems and open discussions as part of positive organisational cultures regarding complaints (Social influences/Opportunity), and a lack of certainty around when to treat issues raised by patients as a formal complaint or informal feedback (Knowledge/Capability).

**Conclusion:**

Our study findings highlight the importance of strong support systems and organisational openness to patient feedback. These findings can be used to design targeted interventions to support more effective responses and enhance patient‐centred approaches to complaints management in healthcare settings.

**Patient and Public Contribution:**

Patient and public involvement (PPI) was integral in this research. The NIHR PRU in Behavioural and Social Sciences had a dedicated PPI strategy group consisting of six external representatives from the patient and public community (Newcastle University, 2024). These six PPI members actively participated in shaping the research by reviewing and providing feedback on all questionnaire items before the data collection. They were actively involved in supporting participant recruitment by advertising this study on their PPI platform, The Voice^R,1^ and through their online social networks. During the analysis stages of the research, preliminary findings were discussed with the PPI group to support ‘sense checking’ and interpretation of the results.

## 1 Introduction

Healthcare complaints provide a mechanism for patients and carers to provide feedback on the quality of healthcare services and their satisfaction with these services. Healthcare complaints can be defined as ‘an expression of dissatisfaction ‐ either spoken or written—that requires a response’ [1]. The complaints process is often stressful for healthcare professionals (HCP) and patients alike [2], but can also provide insights into individual or organisational gaps, oversights or errors in healthcare provision [3, 4]. Therefore, effective complaints resolution can lead to a higher likelihood of patient feedback being utilised to improve future quality of care.

Behaviours exhibited by healthcare professionals during the complaints process (e.g., talking with a patient empathetically) can directly and indirectly influence patient outcomes. For example, HCPs' interactions with patients can influence patients' sense of safety, emotional wellbeing and satisfaction with healthcare [5, 6, 7]. Collaborative healthcare professional‐patient relationships have been associated with better patient outcomes, such as medical adherence [5, 8]. However, certain responses to patient complaints (e.g., fauxpologies, such as ‘I'm sorry you feel…’) can invalidate patients' experiences or even shift blame on to the patient or a specific HCP [3, 9, 10, 11]. In the context of processing healthcare complaints, supporting positive interactions with patients contribute to more constructive resolutions to complaints [12, 13], often resulting in higher satisfaction with care.

Healthcare settings have recently started treating complaints as feedback for quality improvement to limit future harm to patients, and improve experience of healthcare services [3, 14, 15]. This principle is reflected in the UK National Health Service (NHS) Complaints Standards, announced in early 2021 by the Ombudsman [1]. However, despite these efforts at policy level to change complaint responses, change within a complex organisational healthcare setting will require time and effort [16]. Research on the influences on HCP complaint responses can support the identification of strategies to enhance the complaints process and forms the focus of this research. As healthcare provider responses to complaints are behaviours (e.g., talking to a patient empathetically or defensively, apologising or referring patients to appropriate support, passing the information about the complaint to the appropriate place to ensure that improvements are made in a timely manner), behavioural theories and/or models can be used to systematically explore influences on these responses and develop behaviour change strategies to change them.

**Figure 1:**
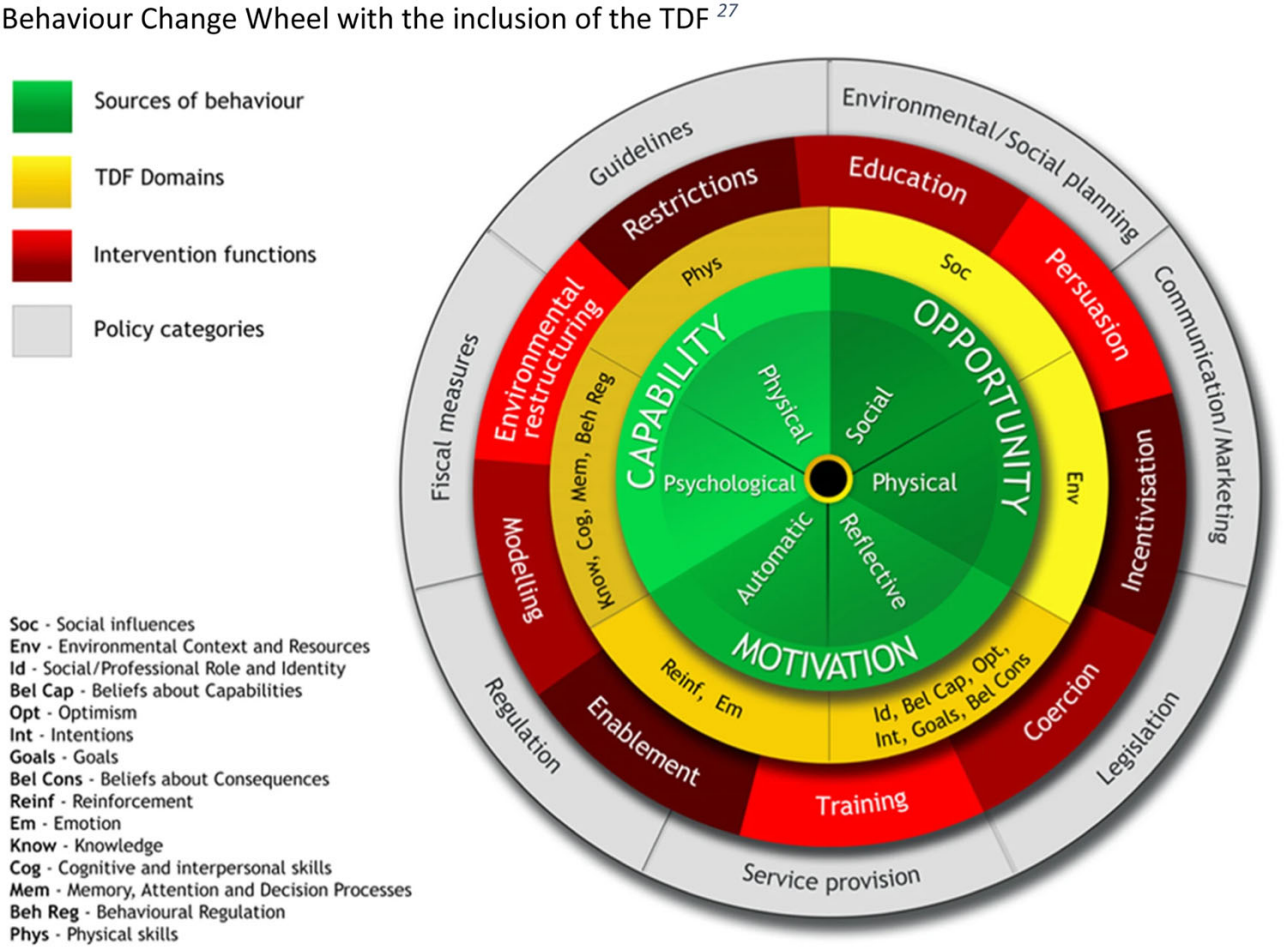
Behaviour change wheel with the inclusion of the TDF [27].

The Theoretical Domains Framework (TDF) [17, 18, 19] and the Capability‐Opportunity‐Motivation‐Behaviour Model (COM‐B) [20] have both been widely used to explore influences on behaviours in healthcare settings, both qualitatively and quantitatively, especially those of healthcare providers [21, 22, 23, 24, 25, 26]. The TDF integrates 128 theoretical constructs taken from 33 psychological theories related to behaviour into 14 domains representing a broad range of influences on behaviour: (1) Knowledge, (2) Skills, (3) Social/Professional Role and Identity, (4) Beliefs about Capabilities, (5) Optimism, (6) Beliefs about Consequences, (7) Reinforcement, (8) Intentions, (9) Goals, (10) Memory, Attention and Decision Processes, (11) Environmental Context and Resources, (12) Social Influences, (13) Emotions, and (14) Behavioural Regulation (see definitions in Supporting Information S1: File 1). The TDF can be further summarised into the constructs of the COM‐B Model, a broader, complimentary model that includes six constructs that influence behaviour: (1) Psychological capability, (2) Physical capability, (3) Physical opportunity, (4) Social opportunity, (5) Reflective motivation and (6) Automatic motivation. The TDF and COM‐B Model have been mapped to and feature in the Behaviour Change Wheel (BCW), which specifies nine broad behaviour change intervention types: Education, Training, Incentivisation, Modelling, Coercion, Persuasion, Enablement, Environmental restructuring, and Restriction and seven policy options: Communication/marketing, Guidelines, Fiscal measures, Regulation, Legislation, Environmental/Social planning, Service provision (see Figure 1) [20, 27]. The mapping facilitates stepwise, systematic intervention development by pointing to different types of behaviour change intervention strategies that are more likely to be effective in addressing different types of COM influences on behaviour.

This study follows on from a systematic review applying the TDF to synthesise knowledge about key influences on HCP responses to complaints, and the wider BCW to identify potential intervention types to target these influences [28]. The results suggested various influences on the responses to complaints, such as ‘Interpersonal skills’ (Skills), ‘Beliefs about the value/consequences of complaints’ (Beliefs about consequences, Intention), ‘Negative emotions—fear of litigation’ (Emotions), and ‘Organisational culture and leadership’ (Social Influences). The review highlighted that responses tend to be immediate (in‐the‐moment) or post‐incident, and response processes were informal (i.e., usually expressed verbally) or formal (i.e., patient feedback about the quality of the care received or a dispute within the healthcare setting) with a provable record of it being made. The review concluded that there was limited published research examining immediate responses to informal concerns/complaints, as most of the literature related to post‐incident responses to formal complaints. Few studies also explicitly reported applying behavioural theories or frameworks to investigate responses to complaints. Moreover, some of the influences mentioned by HCPs (e.g., the availability of informal complaint process) needed to be cross‐checked from patients' and carers' perspectives, as complaint processes are complex, context‐sensitive, and highly dependent on HCPs and service user interactions. Therefore, collecting primary data using behavioural models and frameworks for interviews with HCPs and service users can provide in‐depth insights into these types of responses based on HCPs' and patients' perspectives through a behavioural science lens.

### 1.1 Research Aims

This study sought to explore the factors—the barriers and facilitators—perceived to influence healthcare professionals' responses to complaints from the perspectives of HCPs and service users (i.e., patients and their carers) in the United Kingdom.

## 2 Methods

A qualitative study was conducted using semi‐structured interviews based on the COM‐B Model [20]. Qualitative methodology was used because it can highlight issues of importance to patients and carers (henceforth referred to as ‘patient’ for brevity) about their healthcare experiences [29] and has previously been used to provide insights into HCP views on improving quality of care [30]. This study was approved by the UCL Ethics Committee (20295/003).

### 2.1 Participants and Recruitment

Participants included HCPs and patients or carers with experience of engaging with the complaints process in public secondary and tertiary healthcare settings. Participant inclusion criteria included: (1) work experience in NHS secondary or tertiary settings and having dealt with a complaint within the last 10 years (HCPs), or (2) having made a complaint in either NHS hospital and/or community settings within the last 10 years (patients).

Exclusion criteria for HCPs included: (1) working as primary care or dental care staff (as the regulatory frameworks, the complaints management processes, as well as the types of complaints in these contexts are different from those received in secondary and tertiary care), or (2) receiving complaints related to learning disabilities, mental health, paediatrics, and forensics (as the nature of complaints in these services are highly specialised and therefore, were beyond the scope of this study. Mental health services are often governed by specific legal frameworks and policies that differ from general healthcare services (Mental Health Act 1983). In the United Kingdom, mental health services are subject to additional oversight under the Mental Health Act, and the NHS has dedicated pathways for handling mental health complaints, particularly involving advocacy services like the Independent Mental Health Advocate about involuntary treatment and patient rights [31]. Therefore, these types of complaints were excluded on the basis that merging findings from very different care contexts could dilute the focus of our study, i.e. exploring general complaints in secondary and tertiary care. However, if complaints were not specifically about mental health service delivery [e.g. associated with a chronic physical condition], they were included in our study). Exclusion criteria for patients included: (1) if patients had an active complaint, or (2) if their complaint was related to health services described above, which were beyond the scope of the study. All participants needed to be adults aged over 18 years and residing in the UK.

Participants were invited through adverts for the study posted on social media (e.g., via Twitter, Facebook, LinkedIn), university websites (University College London, Newcastle University), our Patient and Public involvement (PPI) group website (The Voice^R 1^) and other networks, such as the Contact, Help, Advice and Information Network (CHAIN), and via snowballing [32]. As is consistent within an interpretivist paradigm, the aims of the interviews were to explore detailed perspectives of participants taking part, rather than to recruit a pre‐specified number of participants with variably detailed accounts. Therefore, gathering sufficient information to answer the research questions during interviews helped the study team to determine the final sample size. Participant recruitment was completed when the researchers identified no new concepts being discussed during interviews, often termed information power in qualitative research [33].

### 2.2 Materials

Two topic guides were developed based on the COM‐B Model [20], one for the interviews with HCPs and the other for patients and carers (see questions listed in Supporting Information S1: Files 2 and 3). For HCPs, the topic guide questions mainly focused on the factors that influenced their responses to the complaint. For patients and carers, the topic guide was centred on the patients' experience of the complaints process, for example, how they would describe their interaction with the HCP and/or complaints team. The COM‐B Model was selected as a framework to prompt discussions on a wide range of potential influences, with the number of questions on each domain ranging from three to ten. However, no questions were prompted on ‘Physical capability’, as the researchers and PPI members did not consider complaint handling to require any particular physical or musculoskeletal skill (e.g., balance or dexterity) from HCPs.

All six PPI members of the NIHR Policy Research Unit in Behavioural and Social Sciences PPI strategy group provided feedback on the clarity and scope of the topic guide questions. The lead researcher (VA, a senior researcher with expertise in behavioural science and experience in qualitative research interviews) also piloted the questions with a service user, according to which they were refined. Example questions for each COM‐B construct within the topic guides are presented in Table 1.

**Table 1:** Example interview questions based on the COM‐B constructs.

COM‐B construct	Construct description	Example questions from healthcare professional interviews	Example questions from patient interviews
Psychological capability	A capability that involves a person's mental functioning (e.g., understanding and memory)	To what extent do you think you and your colleagues have the necessary skills and training to handle complaints effectively?	Do you think the staff have the necessary skills to handle effectively a complaint?
Physical capability	A capability that involves a person's physique, and musculoskeletal functioning (e.g., balance and dexterity)	N/A	N/A
Physical opportunity	Opportunity that involves inanimate parts of the environmental system and time (e.g., financial and material resources)	Do healthcare personnel have the required time and access to systems to provide the complainant with a regular update and to keep a formal record?	How easy/accessible is it for you/other people to use the system to look for information or if they have to file a report or write a letter/email to complain or to raise concerns?
Social opportunity	Opportunity that involves other people and organisations (e.g., culture and social norms)	What is the culture of complaints handling in your hospital?	Did you get any support when making the complaint? (either from PALS or from any other group)
Reflective motivation	Motivation that involves conscious thought processes (e.g., plans and evaluations)	How confident are you when it comes to complaints management? Do you think complaints handling skills should be part of your role within the hospital?	What changes would you like to see [in response to the complaint process]?
Automatic motivation	Motivation that involves habitual, instinctive, drive‐related and affective processes (e.g., emotions, desires and habits)	How does it feel when you receive a complaint?	How do you feel when your complaint is not being addressed the way you would have hoped?

*Note:* For further COM‐B construct descriptions, see West and Michie [34].

### 2.3 Procedure

Participants who expressed interest in the study were sent an information sheet. After written informed consent was obtained, a researcher (B.G., an MSc‐level researcher with expertise in behavioural science and experience in interviews) conducted the interviews in July and August 2022 on Microsoft Teams or over the phone. Participants were asked questions based on the topic guides to investigate experiences of the complaints process. The interviews lasted between 32 and 90 min and were audio recorded.

### 2.4 Analysis

The interviews were transcribed verbatim by a professional transcription company, and a researcher checked the transcripts for accuracy and removed any identifiable information. Thematic analysis was conducted to reflexively engage with the data and develop themes about the influences on HCPs' responses to complaints [35]. Six researchers were involved in the analysis, V.A., B.G. conducted the main analysis and four other researchers with expertise in behavioural science checked and suggested adaptations to the extracted themes and mapping (P.S., A.M.C., F.L. and A.M.). The analysis process involved inductive coding to develop the themes, followed by a deductive mapping of the generated themes onto COM‐B constructs and the more granular TDF domains [17, 18]. This process broadly consisted of the following steps:

1. Inductive qualitative analysis: To gain familiarity with the interview data [35], two researchers (V.A. and B.G.) independently read through all transcripts and noted down initial codes for the HCPs' and the patients' transcripts.
2. To increase trustworthiness of the analysis [36, 37, 38], all transcripts were double coded by V.A. and B.G. They extracted codes across all transcripts with preliminary labels.
3. The researchers met weekly to discuss discrepancies in their coding to gain a more nuanced understanding of the data [35] and agree on the code labels.
4. Following these discussions, descriptions were drafted for each code alongside the extraction of example quotes (see Table 3 for finalised descriptions). The same two researchers then reviewed and discussed which codes qualified as higher‐level themes or subthemes, creating a preliminary list of themes.
5. To further verify the trustworthiness and clarity of the analysis [36], a third researcher (P.S.) used these themes to code ~20% of transcripts (two randomly selected patient/carer transcripts and two randomly selected HCP transcripts). The lead researcher (V.A.) reviewed this coding, and any lack of clarity about the themes was discussed. The themes, their labels or descriptions were refined where necessary.
6. Deductive analysis mapping onto the COM‐B Model and TDF: Three researchers (V.A., P.S. and A.M.) discussed and agreed on the deductive mapping of the developed themes onto the COM‐B components and TDF domains. The final list of the themes and their mapping were reviewed by the senior team members (F.L. and A.M.C.) and refined accordingly. These were presented visually in a TDF‐COM‐B map, first used by Ojo and colleagues [39]. Participant quotes have been edited with ‘[…]’ for brevity within the text.

## 3 Results

### 3.1 Participant Characteristics

The study included 18 participants (see details in Table 2). Of these, eleven were HCPs who had dealt with a complaint, with some occupying roles related to patient experience, complaints or hospital management. Many had previous experience as clinicians or nurses. The remaining seven participants (*M*_age_: 61 years, age range: 36–76; four females) had been patients (*n* = 3) or patients' carers who complained about patient care (*n* = 4).

**Table 2:** Participant demographic information.

Participant ID	Age range	Gender	Job title
P1	60–64	Female	N/A
P2	60–64	Female	N/A
P3	50–54	Female	N/A
P4	70–74	Male	N/A
P5	75–79	Male	N/A
P6	60–64	Female	N/A
P7	35–39	Female	N/A
HCP1	30–34	Male	Patient Experience Manager
HCP2	40–44	Male	Consultant ENT Surgeon
HCP3	50–54	Female	Regional Complaints Manager
HCP4	45–49	Male	Patient Experience Manager
HCP5	45–49	Male	Consultant Physician
HCP6	50–54	Female	Complaints Manager, PE and Involvement Lead
HCP7	45–49	Female	Complaints Manager
HCP7	55–59	Female	Head of Patient Experience
HCP8	50–54	Female	Complaints Manager
HCP9	45–49	Male	Strategic Complaints Lead
HCP10	57	Male	Lead Nurse in Patient Experience

Abbreviations: HCP, healthcare professional; P, patient.

### 3.2 Themes

**Figure 2:**
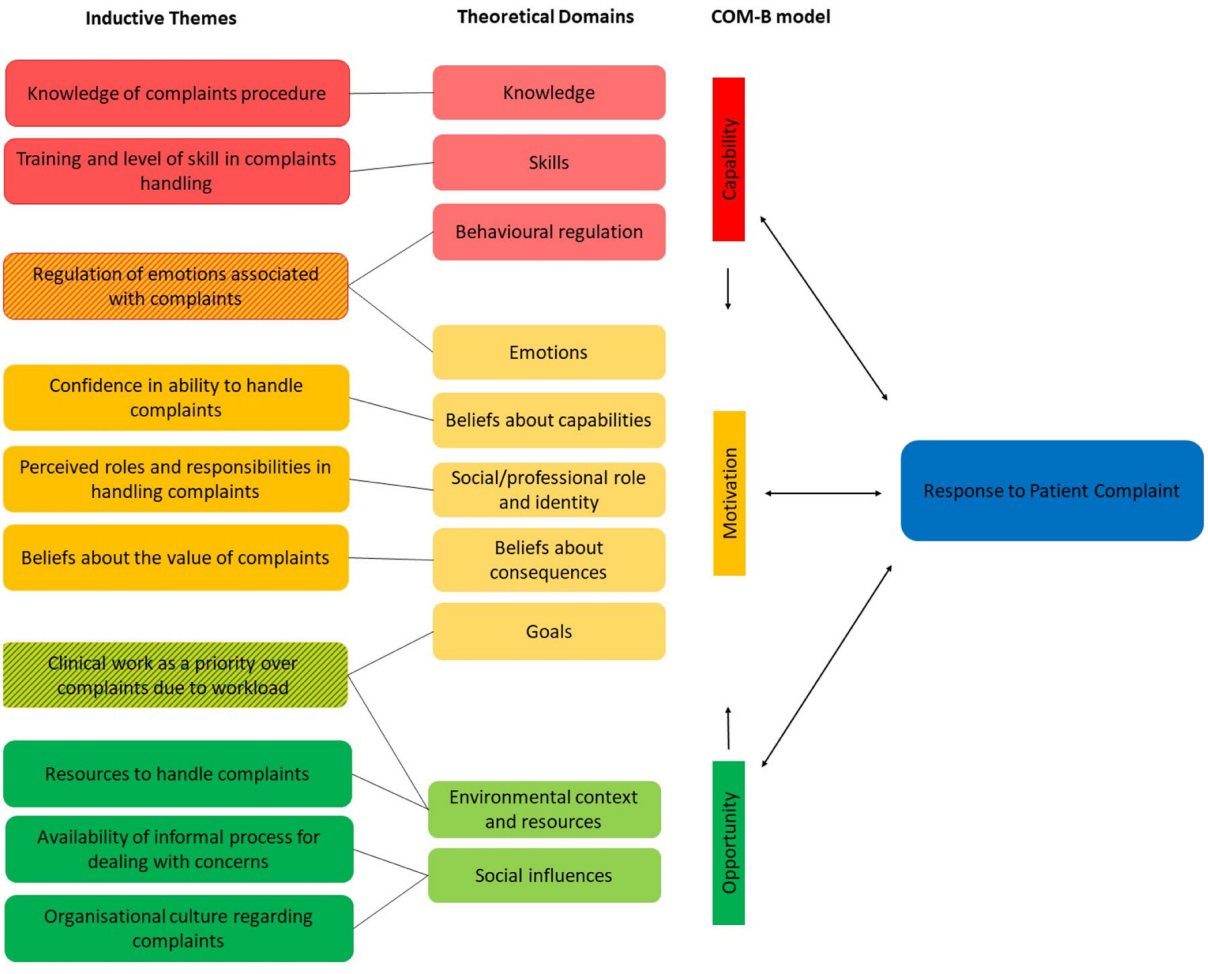
Influences on responses to patient healthcare complaints and their mapping onto TDF domains and COM‐B constructs.

The researchers developed the following 10 themes about the influences on HCPs' complaint response: ‘Knowledge of complaints procedure’, ‘Training and level of skill in complaints handling’, ‘Regulation of emotions associated with complaints’, ‘Confidence in ability to handle complaints’, ‘Perceived roles and responsibilities in handling complaints’, ‘Beliefs about the value of complaints’, ‘Clinical work as a priority over complaints due to workload’, ‘Resources to handle complaints’, ‘Availability of informal process for dealing with concerns’, and ‘Organisational culture’. Of these themes, eight were each organised under one of the following TDF domains: ‘Knowledge’, ‘Skills’, ‘Beliefs about capabilities’, ‘Social/professional role and identity’, ‘Beliefs about consequences’, ‘Social influences’ and ‘Environmental context and resources’. Two themes were mapped each onto two different TDF domains, with one being mapped onto both ‘Emotions’ and ‘Behavioural regulation’, and the other being mapped onto ‘Goals’ and ‘Environmental context and resources’. Figure 2 provides an overview of these themes.

From the 10 themes, the researchers identified 6 themes in both HCP and patient interviews, while 4 themes were only identified in HCP interviews. These will be presented together to provide a better understanding of the themes from both perspectives. Table 3 presents theme and subtheme descriptions alongside illustrative supporting quotes.

**Table 3:** The themes about the influences on healthcare professionals’ responses to complaints, theme descriptions, example quotes and mapping onto TDF domains and COM‐B constructs.

Themes	Theme description	Example quotes and related narrative	TDF domains	COM‐B constructs	Participant group
1. Knowledge of complaints procedure	Participants expressed HCPs being aware of the complaint procedure and guidelines and where to find information about complaints if they need it.	‘We're all still governed by the 2009 Local Health Authority and Social Services Complaints Regulations so that's pretty much the legal framework that everybody has to follow which has a mixture of […] requirements in terms of the way you have to do this’. HCP10, Strategic Complaints Lead	Knowledge	Psychological capability	HCPs and patients
2. Training and level of skill in complaints handling	Participants expressed that HCPs had different skills levels in dealing with complaints and the training available to improve their skills varied.	‘There is no sort of national complaints handling training or no requirement for a qualification currently and that's something that's been discussed several times over the years’. HCP3, Regional Complaints Manager ‘You have to be quite open‐minded and have good listening skills’. HCP 9, Complaints Manager	Skills	Psychological capability	HCPs and patients
3. Regulation of emotions associated with complaints	Participants expressed that regulating negative emotions associated with complaints enabled more constructive approaches to such complaints.	‘Inevitably, you'll probably be doing something else when you just open an email and… then you suddenly get annoyed. The wise thing to do with that is to close it and come back to it when you've got time and respond objectively’. (HCP 2, Consultant ENT Surgeon)	Emotions; Behavioural regulation	Automatic motivation; Psychological capability	HCPs
4. Confidence in ability to handle complaints	Participants expressed that HCPs had different confidence levels in handling complaints based on their training and/or experience.	‘Years of experience gives more confidence to responding to complaints’. HCP5, Consultant Physician	Beliefs about capabilities	Reflective motivation	HCPs
5. Perceived roles and responsibilities in handling complaints	Participants expressed that the perceived roles and responsibilities could influence how HCPs responded to complaints.	Perceived role of HCPs in handling complaints: Participants expressed that HCPs interacting with patients and those in the complaints’ department were responsible for handling complaints, but also that the role of complaints officers could present challenges to balancing the interests of different parties in a complaint process. ‘Anybody in patient facing roles […] needs to be familiar with these [complaint] processes and avenues and tactics for resolution, but yes certainly in my role, I need to know that’. HCP4, Patient Experience Manager	Social/professional role and identity	Reflective motivation	HCPs and patients
6. Beliefs about the value of complaints	Participants noted that HCPs should value and view complaints as feedback to improve patient care.	‘I think there's something about genuinely valuing complaints as an opportunity to do something differently or better, and then being responsive to what those complaints say. So there's something about a) how to deal with it in a humanistic way, b) what the value is in dealing, using complaints and there's something about the ability of the person to be of a service to be responsive to that, so rather than coming from a point of defence, coming from a point of acceptance and actually accept that regardless of the reasons they've had a bad time of it, so let's try and see if from their perspective’. HCP 4, Patient Experience Manager	Beliefs about consequences	Reflective motivation	HCPs
7. Clinical work as a priority over complaints due to workload	Participants expressed that clinical work was considered a priority over handling complaints for staff and the organisation as a whole, often related to the high workload staff and organisations have.	‘When complaints come in, I deal with them immediately, but we know that people don't do… that automatically because of the pressures… in the hospitals that, that you will have read about […] And so that's why they're [complaint responses] not seen as important […] that's […] probably one of the key reasons why they [complaints] haven't historically been responded to in a timely manner’. HCP 11, Lead Nurse in Patient Experience	Goals; Environmental context and resources	Reflective motivation; Physical opportunity	HCPs
8. Resources to handle complaints	Participants stated that there seemed to be variable cultures and available resources to address complaints within and across organisations.	Time and resources required to respond to complaints. ‘It probably is a time and resource… I'm sure though that there are things that could be done for sure… a specific person just for staff support within a complaints team or within a wider patient experience team would be really good […] but it comes back to time and money’. HCP 7, Complaints Manager	Environmental context and resources	Physical opportunity	HCPs and patients
9. Availability of informal process for dealing with concerns	Participants indicated variability in whether hospitals had an informal process for handling complaints, which could be useful for quicker and/or less bureaucratic responses to complaints.	‘There's a fundamental problem in that there's no process for raising concerns and for teaching people how to deal with concerns. If the only process available is to make a formal complaint, I think that's a fundamental problem. So given that that's the case, I don't think that staff are then able to empathise with patients […] so they're not allowed to because now we're in a defensive mode where we need to protect the organisation’. P2	Social influences	Social opportunity	HCPs and patients
10. Organisational culture	Participants stated that there seemed to be variable cultures to support the resolution of complaints.	Participants expressed that there was mixed social support for handling complaints, often depending on the organisation's leadership. ‘It depends on your line manager. So, that does depend on your line manager and that is definitely the answer for here. However, the one I can think about I would say not very well supported, no […] I've seen it done well with a line manager and I've seen it done really poorly, but I think on the whole done poorly’. HCP5, Consultant Physician	Social influences	Social opportunity	HCPs and patients

### 3.3 Theme 1: Knowledge of Complaints Procedure

#### 3.3.1 TDF Domain: Knowledge/COM‐B Construct: Psychological Capability

HCPs generally expressed knowing complaints procedures and where they could learn more about these procedures if needed. Such knowledge supported them in responding to complaints, finding out more information about specific complaint procedures, and/or signposting patients or carers to relevant information. Knowledge of formal complaint guidelines was noted as being particularly important when responding to healthcare complaints. For instance, as one participant explained: ‘There are… many policies, guidelines, and regulations that you have to adhere to when you're responding to a healthcare complaint so those would always come into play… whenever you receive a complaint’ (HCP7, Complaints Manager). However, HCPs also highlighted uncertainty on when to treat a concern raised by patients/carers as a ‘complaint’ or ‘feedback’, as there is no national guidance on this: ‘There is still that general unwritten rule with no guidance… around what's a complaint and what could be treated as a concern or feedback’. (HCP10, Strategic Complaints Lead).

### 3.4 Theme 2: Training and Level of Skill in Complaints Handling

#### 3.4.1 TDF Domain: Skills/COM‐B Construct: Psychological Capability

Participants explained that HCPs differed in their training and level of skills for handling complaints. There were different types of training available regarding the complaint handling process within and across hospitals, ranging from surface‐level customer service training to in‐depth complaints training. One participant indicated that there was some training provided in their trust around ‘customer service’ that was available to everyone. As a senior staff member, HCP10 was provided additional training from an outside organisation, including topics around ‘how to investigate the complaint… mediate and negotiate within the complaints and… respond to a complaint’ (HCP10, Lead Nurse in Patient Experience). Several HCPs who worked as complaints officers also mentioned receiving training when starting their positions. Therefore, senior management and complaints officers seemed to receive more in‐depth training, while other staff seemed to be provided with more surface‐level training.

Other HCPs expressed that their organisation did not have any formal or mandatory training, with HCP3 (Regional Complaints Manager) pointing out that there is no ‘national complaints handling training or no requirement for a qualification’. Some HCPs said that training could help them to develop better communication skills and deal with the emotional side of complaints. However, others questioned the need for mandatory training, as ‘there's enough mandatory training and people… don't take mandatory training very seriously’. (HCP2, Consultant ENT Surgeon). Instead, it was considered more useful to provide staff a simple overview of complaint types and support HCPs within the organisation (e.g., with legal representatives that they can speak to).

Both HCPs and patients seemed to view interpersonal and communication skills as key for handling complaints. For example, patients and carers appraised open communication favourably: ‘[the HCP] was friendly, she wasn't, you know, defensive. She immediately reassured me that she had looked into this [complaint]’. (P7). HCPs' abilities to listen and be receptive to feedback were considered as particularly important when interacting with patients. Several HCPs also highlighted the need to communicate in a friendly and non‐bureaucratic way with patients and carers, but also other HCPs who are dealing with a complaint. For instance, one participant expressed: ‘It's easy to hide behind the bureaucracy of a process, when actually it needs a human being, a human intervention to phone, go see somebody… we've got to be friendlier and more human about our complaints processes… to our staff who are investigating them, but also to the people who are on the receiving end’. (HCP4, Patient Experience Manager).

### 3.5 Theme 3: Regulation of Emotions Associated With Complaints

#### 3.5.1 TDF Domains: Emotions; Behavioural Regulation/COM‐B Construct: Automatic Motivation; Psychological Capability

HCPs and patients generally agreed that complaint processes are stressful and can negatively affect mental health. Some HCPs noted that these negative emotions could lead to more charged or defensive interactions during the complaint process, especially relating to worries about being blamed as part of the complaint process. However, regulating their emotions helped them approach complaints more constructively. Several HCPs described feeling upset in response to receiving a complaint, but distancing oneself from the initial negative emotions enabled a more objective response: ‘You feel like you've let somebody down… I suppose you go through those emotions of that's wrong, that's unfair… so almost like that grief cycle … and depending how reflective you are as an individual… you might give it some time and come round and go actually, what could I have done’. (HCP4, Patient Experience Manager).

For people working in complaints related roles, one participant noted that sometimes ‘you might feel that your compassion fatigue is kicking[in]’ (HCP10, Lead Nurse in Patient Experience). In such cases, the HCP suggested that people need to take a ‘time out’ or ‘step back from this particular element of the role or this particular case’. Otherwise, they might end up approaching cases not as compassionately as they normally would. Therefore, HCPs taking time to process and regulate their emotions associated with complaints was linked to more positive complaint responses.

### 3.6 Theme 4: Confidence in Ability to Handle Complaints

#### 3.6.1 TDF Domain: Beliefs about Capabilities/COM‐B Construct: Reflective Motivation

Several HCPs mentioned that confidence levels in handling complaints often related to the training staff received or their experience in handling complaints. For instance, HCP5 (Consultant Physician) explained ‘experience gives more confidence to responding to complaints’, which could lead to more effective communication when managing complaints. HCP9 (Complaints Manager) pointed out that new complaints' officers usually ‘feel confident’ once they had ‘a proper training programme that covers not only the processes but also… some practical elements’, such as observing more experienced training officers. When staff have lower ‘confidence levels’ in dealing with complaints, HCP5 pointed out that they felt they needed more support from their colleagues or the complaints' department.

### 3.7 Theme 5: Perceived Roles and Responsibilities in Handling Complaints

#### 3.7.1 TDF Domain: Social/Professional Role and Identity/COM‐B Construct: Reflective Motivation

HCPs' perceptions of their own or others' roles and responsibilities in handling complaints influenced their subsequent responses to complaints.

*Subtheme 5.1: Perceived role of HCPs in handling complaints*

HCPs generally agreed that handling complaints was a responsibility of any HCP that interacts with patients and/or helps manage complaints in a healthcare organisation. Several participants noted challenges in balancing different parties' interests to resolve a complaint, particularly with complaints staff being based in the same organisations as the people that were complained about. HCP 1 explained that their role in the complaints' department required them to consider both the needs of their colleagues and the patients. However, some patients expressed concerns regarding the fairness of the complaint process because of the relationship between the people being complained about and those helping resolve complaints. Two participants noted that having an independent investigator could make the complaints processes fairer: ‘The whole system doesn't work because there is no independent investigators… There should be no connection at all, financial connection, or contribution from the hospital to the investigation process’ (P6).

*Subtheme 5.2: Openness and transparency/duty of candour*

Several participants explained that being open and transparent was an important aspect of HCPs' roles when dealing with complaints. HCP3 (Regional Complaints Manager) stated that ‘duty of candour and being open and transparent’ are key when communicating with patients or carers. Therefore, when HCPs perceived openness and transparency as part of their responsibilities, they tried to communicate as transparently as they could with patients and carers. Moreover, this perceived responsibility was discussed in relation to a willingness to receive feedback and learn from mistakes. HCP9 (Complaints Manager) indicated that HCPs in their organisation were ‘open and transparent’ and ‘admit failings’, with ‘the most important part [being] the learning from it’. One participant (P7) expressed appreciating an HCP for responding to their feedback honestly and acknowledging mistakes, but also explained that a more formal legal complaint process might have made it difficult for the HCP to communicate as openly.

*Subtheme 5.3: Perceived role of Patient Advice and Liaison Services (PALs) in handling complaints*

Several HCPs mentioned that the perceived role of Patient Advice and Liaison Services (PALs) in addressing complaints varies across different hospitals. In some hospitals, HCP9 (Strategic Complaints Lead) suggested that the role of PALs included facilitating ‘informal resolution[s]’ to an issue (e.g., communicating with the patient informally), whereas there were other hospitals ‘where pretty much everything… is automatically entered into the complaints process’. The participant further underlined that there is ‘no national framework around how PALS should look’, leading PALS to be utilised differently across hospitals.

### 3.8 Theme 6: Beliefs about the Value of Complaints

#### 3.8.1 TDF Domain: Beliefs about Consequences/COM‐B Construct: Reflective Motivation

Many HCPs said that it was important to view complaints simply as feedback about patient care, and thereby appreciate opportunities to learn from feedback to improve healthcare services. For instance, one participant expressed: ‘I think we should encourage people to complain, so we can learn from it, if you know, you know’ (HCP5, Consultant Physician). Similarly, HCP7 (Complaints Manager) explained that they are a ‘believer that a complaint is just feedback… people like positive feedback and they don't like negative feedback as much, but you can learn from both…’. Therefore, by framing complaints as feedback, HCPs seemed to respond to complaints more pragmatically and show willingness to learn from them.

### 3.9 Theme 7: Clinical Work as a Priority Over Complaints Due to Workload

#### 3.9.1 TDF Domains: Goals; Environmental Context and Resources/COM‐B Constructs: Reflective Motivation; Physical Opportunity

HCPs reported feeling pressure to prioritise clinical work and other responsibilities over handling complaints on both an individual and an organisational level, especially considering the resources available within the NHS. For instance, HCP4 stated: ‘Workloads are enormous in the NHS… general workloads, clinical workloads are enormous, people are getting sicker, older. So, we're getting busier, staff are getting fewer… so it's a perfect storm that… complaints aren't, won't be a priority… against the clinical work’. (HCP4, Patient Experience Manager).

The time available to HCPs to spend on handling complaints seemed to vary based on their roles. Particularly HCPs working as clinical staff expressed challenges in spending time on complaints, even if they considered handling complaints to be important. One participant said: ‘If your job is dealing with complaints, you will have time to deal with complaints. If your role is as a consultant who is trying to deal with a complaint, on top of this job plan, which I am doing, I don't have a lot of time, however it's important…’ (HCP5, Consultant Physician).

### 3.10 Theme 8: Resources to Handle Complaints

#### 3.10.1 TDF Domain: Environmental Context and Resources/COM‐B Construct: Physical Opportunity

Having the time, responses and available means of resolving complaints informally influenced HCPs' behavioural responses to patient healthcare complaints.

Participants underlined that the time and resources available to HCPs influences how quickly and thoroughly they can address complaints. One participant expressed that having a support staff within the patient experience (e.g., ‘staff liaison person’) could help HCPs handle complaints better, but the availability of such staff required ‘time and money’ (HCP7, Complaints Manager). Some HCPs highlighted challenges in responding to complaints, when there is a high number of patients using a healthcare site. For instance, HCP6 (Complaints Manager) explained that their ‘response rate is low [in the complaints' office], mainly due to the complexity of them and because we're a large tertiary site’.

Additionally, having an easily accessible electronic record of complaints was considered to enable quicker responses to complaints, and the record systems used seemed to vary across healthcare organisations. In one organisation HCP7 (Complaints Manager) had worked at, ‘information… tended to be spread across a few [electronic] systems’, making complaints ‘time‐consuming to respond to properly’. A particular challenge for HCPs was when their organisation had paper‐based healthcare records instead of electronic ones.

### 3.11 Theme 9: Availability of Informal Process for Dealing With Concerns

#### 3.11.1 TDF Domain: Social Influences/COM‐B Construct: Social Opportunity

Participants suggested that the availability of informal processes to address concerns varied across organisations. HCP4 (Patient Experience Manager) explained that the PALS in their previous organisations had a route that allowed them to ‘resolve [some complaints] easier and quicker’ rather than going through a ‘more bureaucratic process’. Patients expressed wanting an option for resolving complaints through informal processes, particularly to address immediate health issues. One participant also noted that they knew they could escalate their complaint if they were unhappy with the informal process (P7). Another participant (P2) linked the lack of a viable informal process for resolving complaints to HCPs having to be in a ‘defensive mode where… [they] need to protect the organisation’.

### 3.12 Theme 10: Organisational Culture Regarding Complaints

#### 3.12.1 TDF Domain: Social Influences/COM‐B Construct: Social Opportunity

The availability of organisational or peer support, and organisational culture acted at times as influences of HCPs' behavioural responses to complaints.

*Subtheme 10.1: Organisational and peer support for handling complaints*

HCPs suggested that there was some organisational and peer support (e.g., legal support and support from managers or colleagues) available for staff handling complaints. However, the level of support seemed to vary across organisations. HCP8 (Head of Patient Experience) emphasised the need for ‘formalising support’. Some examples of formal support were mentioned, such as having weekly debriefs to raise ‘difficult conversations or anything that they've found upsetting and distressing’ (HCP3, Regional Complaints Manager). The availability of support for staff was often linked to management, both line managers and more senior management. Senior management were noted as having a key role in making sure that there are ‘mechanisms in place to make sure people are supported’ (HCP2, Consultant ENT Surgeon).

*Subtheme 10.2: Organisational defensiveness regarding complaints*

HCPs and patients seemed to have mixed views on whether healthcare organisations' culture regarding complaints enabled or interfered with appropriately handling complaints. HCPs sometimes expressed that their organisation had a defensive position and, in some cases, a blame culture when it came to complaints. For instance, HCP5 (Consultant Physician) said: ‘there's still a [tendency]… of potentially trying to shift the blame back onto the patient’ or others within the organisation. A patient also noted that the response to their complaint suggested a defensive organisational position: ‘The impression that I got from the letter… was we must at all costs avoid litigation… but that was never my intention… to litigate, all I wanted was that the Chief Executive would look at all the […] failures that had happened over a long period of time’. (P2).

*Subtheme 10.3: Culture depends on the chief executive/senior staff*

Several HCPs pointed out that the organisational culture depended on leadership within the organisation, and management could influence positive cultural changes. For instance, HCP4 (Patient Experience Manager) explained ‘where there's good leadership there will be good governance and good sharing of learning […] organisationally’. Similarly, HCP9 (Complaints Manager) noted that a previous chief executive ‘did lots of work to… change that [blame] culture and I think it has shifted’.

Patients expressed that some HCPs within organisations seemed to have less power to resolve issues compared to others. In particular, junior staff and sometimes nonclinical staff were considered to have less authority to openly talk about complaints or make changes: ‘The complaints manager did everything he could within his power. But […] the people who he was talking to are the people who are employing him. And he was a junior to them. How can he possibly challenge them?’ (P6).

Therefore, there was a perceived power imbalance in the person handling the complaint and clinical staff who would be responsible for implementing changes in response to it.

## 4 Discussion

Understanding HCP behavioural responses to patient healthcare complaints can be utilised to enhance positive outcomes for patients, HCPs and healthcare organisations. This qualitative study explored the influences on effective complaints resolution in relation to HCPs' responses from both HCPs' and patients' perspectives, analysed using the COM‐B and TDF. Evidence from this study highlighted six influences on responses to complaints identified by patients and HCPs, including knowledge, skill, perceived roles in handling complaints, available informal routes to resolve complaints, resources and organisational culture. Additionally, HCPs identified four unique influences, including the importance of emotional regulation, beliefs about capability to handle the complaint, beliefs about a complaint's value, and clinical work as a priority over complaints handling.

Participants in this study highlighted communication skills as important for the effective resolution of patient healthcare complaints (Theme 2: ‘Training and level of skill in complaints handling’). Previous research has suggested that context specific training for HCPs can help support communication skills development (e.g., asking open questions, active listening and sharing medical notes with patients [40]), and support HCP feelings of self‐efficacy, resulting in improved interactions with patients [41]. In fact effective communication skills may help prevent healthcare complaints in the first place [42].

A prominent theme that came out was healthcare professionals' need to regulate emotions (including high levels stress) associated with the complaint process (Theme 3: ‘Regulation of emotions associated with complaints’). Emotions, particularly fear of litigation, are linked to complaints not only because they can strain the patient‐HCP relationship, but also because complaints can lead to legal consequences (such as impacting HCPs' licence to practice) [27]. Therefore, emotional regulation strategies are key skills identified by participants in this study that can benefit the complaints process by increasing empathy and reducing emotional burnout in healthcare settings [43, 44], including cognitive re‐evaluation techniques to help reappraise and reframe the situation from critical to constructive [45].

Previous research suggests that a high proportion of patients expect a fair and impartial process after lodging a healthcare complaint [46]. To ensure this, there are some mechanisms in place for external and independent investigations for unresolved formal complaint cases in the United Kingdom, namely by the Parliamentary Health Service Ombudsman, which operates independently from the NHS [47] However, many participants in our study noted concerns around the objectivity of the existing complaints process within the NHS hospitals or Trusts, considering that HCPs who investigated complaints were based in the same organisation as those being complained about (Theme 5: ‘Perceived roles and responsibilities in handling complaints’). This echoed findings from our recent systematic review [28] where others have identified inherent contradictions within the complaint manager roles, whereby complaints officers are required to investigate patient complaints made against colleagues in the same organisation [48]. Although the group of participants in this study were self‐selected, all HCPs perceived complaints handling as an integral part of their responsibilities and viewed complaints as learning opportunities. Such perspectives could support the uptake of future patient safety policies, such as the new NHS Complaints Standards, although further work is needed to investigate whether these perspectives are shared more widely by other HCPs.

Several key behaviours that were valued by patients and carers included receiving an apology from the HCP or service provider, as well as an assurance that care quality would improve, rather than pursuing routes such as litigation. Patients and carers seemed to prefer accessing a system that is not geared towards an immediate defence or litigation. Participants found that informal processes often led to more timely responding to complaints, reducing the frustration and stress associated with long waiting periods. Informal processes to raise concerns were also noted as potentially supporting open discussions about issues and enabling patients and HCPs to resolve complaints more cooperatively (Theme 9). However, in more complex cases, HCPs stated that additional time was necessary to thoroughly investigate and properly resolve the issues. Consistently, some patients also expressed a preference for receiving detailed explanations and suggestions for service improvement where necessary, rather than a brief phone call or apology letter that left the issue unresolved. However, participants reported that there were no clear guidelines for HCPs to decide whether an informal route to resolving complaints was appropriate. In addition, despite the potential benefits of less formal routes of resolving complaints, the results highlighted that such routes were unavailable in some organisations (Theme 9: ‘Availability of informal process for dealing with concerns’).

### 4.1 Implications for Practice

Drawing on the BCW approach [27], several intervention strategies can be selected to address the influences for more efficient and constructive complaint responses (Table 4). Taken together, interpersonal skills and emotions can be supported through the intervention types ‘Education’ and ‘Training’. For instance, existing education and training can be refined to focus on HCP communication skills (e.g., active listening) and behavioural/emotional regulation strategies (e.g., self‐monitoring, action planning) when receiving patient feedback. However, as highlighted by participants in this study, where clinical work was seen as a key priority in the face of high work pressures and restrictions on time, future training revisions would need to be balanced in light of existing workloads. Moreover, such training would need to be piloted within healthcare settings to evaluate acceptability, feasibility and effectiveness.

**Table 4:** Examples of intervention strategies to support the area of patient complaints.

Themes	TDF domains	COM‐B constructs	Examples of interventions strategies, based on the findings and research literature
1. Knowledge of complaints procedure	Knowledge	Psychological capability	Increase knowledge of procedures such as issuing guidelines and hospital newsletters. Provision of educational materials and training sessions to staff on the importance of complaints handling and the correct procedures to follow.
2. Training and level of skill in complaints handling	Skills	Psychological capability	Further training and guidance on communication skills, such as asking open questions, active listening and sharing medical notes with patients [40].
3. Regulation of emotions associated with complaints	Emotions; Behavioural regulation	Automatic motivation; Psychological capability	Supporting strategies to avoid emotional burnout, such as cognitive re‐evaluation techniques to help reappraise the situation from critical to constructive [45].
4. Confidence in ability to handle complaints	Beliefs about capabilities	Reflective motivation	Managers offering emotional support and encouragement to healthcare staff when dealing with complaints
5. Perceived roles and responsibilities in handling complaints	Social/professional role and identity	Reflective motivation	Continuing to strengthen workplace culture of conceptualising complaints as constructive.
6. Beliefs about the value of complaints	Beliefs about consequences	Reflective motivation	Support constructive beliefs regarding complaints, strengthening a learning perspective regarding complaints, for instance by: facilitating regular safe spaces for HCPs to discuss complaints, emotions associated with them and potential learnings.
7. Clinical work as a priority over complaints due to workload	Goals; Environmental context and resources	Reflective motivation; Physical opportunity	Managers should clearly communicate the importance of complaints handling as a priority task to all staff members, as well as allocate specific time slots in staff schedules dedicated to complaints handling, thus making it a regular part of their workload.
8. Resources to handle complaints	Environmental context and resources	Physical opportunity	Support organisations to transition to electronic systems or improve the usability of such systems to allow HCPs to easily access patient information relevant to a complaint.
9. Availability of informal process for dealing with concerns	Social influences	Social opportunity	Creating awareness among HCPs regarding informal complaints resolutions, such as issuing an apology and giving assurance that care quality will be improved in future.
10. Organisational culture	Social influences	Social opportunity	Strategies could include reframing organisational policies and practices (e.g. ‘open team discussions’ about complaint management) to highlight the importance of complaints as a quality improvement function within the organisation

As some hospitals seemed to lack informal conflict resolution processes and HCPs seemed to require more guidance on when to initiate such processes, the intervention types ‘Environmental restructuring’, ‘Education’ and ‘Enablement’ can be applied to create official routes and guidance for informal processes within hospitals. Clearer stepwise guidance could support HCPs in deciding when informal resolution processes would be appropriate. Antonopoulou and colleagues [28] suggested that assessing patients' expectations for effective resolution could inform the resolution planning. Accordingly, within guidance given to HCPs, it would be useful to integrate a step to assess patients' expectations before deciding on a formal or informal resolution process.

At organisational level, timely and effective complaints management can be facilitated with accessible electronic systems that can help speed up the management processes as well as enable complaints managers to provide complainants with a regular update on the stage of their complaint during the process. The intervention type ‘Environmental restructuring’ can be applied to address the availability of accessible electronic complaint systems. Accordingly, refining the complaint management systems to be centralised and electronic could simplify accessing information relevant to complaints and thereby responding to these in an appropriate and timely manner. Importantly, this can support the organisation to aggregate complaints data and learn from formal complaints processes. Although we fully subscribe to the idea that learning within complex systems is not a linear process as many factors within the system interact to either create learning opportunities or negative feedback loops that hinder learning, therefore a systemic approach would be best placed to provide direction for successful organisational learning. It is noteworthy from findings from a realist review that development of a standardised taxonomy for reporting of complaints could help not only to categorise severity of complaints upon receive, but the data generated could serve to represent patient voices on a more collective level to influence systemic change [47]. Therefore, more centralised systems could also support more systematic learning from complaints. However, digital transformations in healthcare organisations can be extremely complex and costly [49], making further considerations on how this strategy could be implemented crucial.

### 4.2 Strengths and Limitations

A strength of this research is the exploration of a topical area of healthcare service provision. Our study addresses an important gap in the literature by examining healthcare professionals' behavioural responses to patient complaints. While prior research has primarily focused on the types and content of complaints and their outcomes, there has been limited investigation into the behaviours and psychological processes that shape professionals' responses. [50, 51] By applying behavioural frameworks, our study provides valuable insights into the mechanisms driving these responses, offering new perspectives on how complaints can be managed more effectively. A key finding with important practice implications is that many HCPs in the study already viewed complaints as ‘feedback’ and complaint handling as part of their job role, which may account for the themes identified reflecting positive attitudes towards patient healthcare complaints. This contrasts with previous studies where complaints were viewed and managed as a separate and lower status activity from core clinical duties [2, 52, 53]. However, due to the type of ethical approval (from a university ethics committee and not NHS ethical approval), there were constraints related to advertising the study, which may have prevented the elicitation of a wider range of viewpoints on patient complaints. Only adverts on social media and social networks (not on NHS professional networks) were permitted, which may have limited the reach of those recruited.

Importantly, this study did not specifically examine complaints related to mental health, cognitive impairments affecting informed consent, or social care arrangements, as these involve different complaint mechanisms and distinct influences and thus were beyond the scope of this study. These areas merit further exploration in future research.

## 5 Conclusions

The handling of patient complaints is fraught with complexity and is often emotionally taxing, for both patients and for healthcare professionals. Complaints management requires ongoing support on the part of healthcare organisations to ensure HCPs are equipped to resolve complaints effectively. The evidence presented here extends our understanding of this topic area offering evidence‐based and theoretically driven considerations for the future development of policies, guidance and support offered to HCPs to help enable the management of patient safety in their work, including further training opportunities and transparent processes that facilitate trust. Training offered on how HCPs respond and navigate complaints should draw on case studies of best practice and lessons learned from past experiences within the organisation.

## Author Contributions

**Vivi Antonopoulou:** conceptualisation, methodology, investigation, validation, formal analysis, visualisation, project administration, writing–original draft, writing–review and editing, data curation. **Paulina M. Schenk:** methodology, investigation, validation, formal analysis, visualisation, project administration, writing–original draft, writing—review and editing. **Alison R. McKinlay:** methodology, investigation, validation, formal analysis, visualisation, project administration, writing—original draft; writing—review and editing. **Paul Chadwick:** conceptualisation, writing—review and editing, methodology, funding acquisition. **Carly Meyer:** conceptualisation, methodology, investigation, data curation, writing—review and editing, project administration, validation. **Beckie Gibson:** data curation, methodology, investigation, writing—review and editing, project administration. **Falko F. Sniehotta:** conceptualisation, methodology, investigation, funding acquisition, writing—review and editing, resources. **Fabiana Lorencatto:** conceptualisation, methodology, investigation, funding acquisition, writing—review and editing, supervision, resources. **Ivo Vlaev:** conceptualisation, methodology, writing—review and editing, funding acquisition, resources. **Angel M. Chater:** conceptualisation, investigation, supervision, methodology, resources, writing—review and editing, funding acquisition.

## Ethics Statement

This research was reviewed and approved by the UCL Ethics Committee, ref: 20295/003.

## Conflicts of Interest

The authors declare no conflicts of interest.

## Supporting information

Supporting Information.

## Data Availability

Data and materials can be made available from the corresponding author upon reasonable request.
