# Supplementary material for: Healthcare Professionals' Responses to Complaints: A Qualitative Interview Study With Patients, Carers and Healthcare Professionals Using the Theoretical Domains Framework and COM‐B Model
Source: Health Expect. 2024 Dec 8;27(6):e70118. doi: 10.1111/hex.70118 (PMC11625874; doi:10.1111/hex.70118)
Supplement: Supplementary file 1 — Supporting Information. [file HEX-27-e70118-s001.docx]

Supplementary files

Supplementary file 1 – Definition of TDF subdomains^52^

| **No** | **Domain label** | **Domain definition** |
| --- | --- | --- |
| 1 | Knowledge | An awareness of the existence of something |
| 2 | Skills | An ability or proficiency acquired through practice |
| 3 | Social/Professional Role and Identity | A coherent set of behaviours and displayed personal qualities of an individual in a social or work setting |
| 4 | Beliefs about Capabilities | Acceptance of the truth, reality, or validity about an ability, talent, or facility that a person can put to constructive use |
| 5 | Optimism | The confidence that things will happen for the best or that desired goals will be attained |
| 6 | Beliefs about Consequences | Acceptance of the truth, reality, or validity about outcomes of a behaviour in a given situation |
| 7 | Reinforcement | Increasing the probability of a response by arranging a dependent relationship, or contingency, between the response and a given stimulus |
| 8 | Intentions | A conscious decision to perform a behaviour or a resolve to act in a certain way |
| 9 | Goals | Mental representations of outcomes or end states that an individual wants to achieve |
| 10 | Memory, Attention and Decision Processes | The ability to retain information, focus selectively on aspects of the environment and choose between two or more alternatives) |
| 11 | Environmental Context and Resources | Any circumstance of a person's situation or environment that discourages or encourages the development of skills and abilities, independence, social competence, and adaptive behaviour |
| 12 | Social influences | Those interpersonal processes that can cause individuals to change their thoughts, feelings, or behaviours |
| 13 | Emotions | A complex reaction pattern, involving experiential, behavioural, and physiological elements, by which the individual attempts to deal with a personally significant matter or event |
| 14 | Behavioural Regulation | Anything aimed at managing or changing objectively observed or measured actions |

Supplementary file 2 - Topic guide for healthcare professionals

| **COM-B Construct** | **Questions** |
| --- | --- |
| A.1 Psychological capability | 1. Talk me through the processes of complaint handling in your organisation? |
|  | 1. Is it part of your role to know these processes? |
|  | 1. How do you decide how to respond? What decisive factors do you take into account? |
|  | 1. To what extent do you think you and your colleagues have the necessary skills and training to handle complaints effectively? |
|  | 1. What type of training you thinking would be required to equip staff with the necessary skills to respond efficiently to complaints? |
|  | 1. Based on your experience, how do you explain the outcome of a complaint to the complainant? |
|  | 1. How open and transparent do you think this process is? |
|  | 1. Are you aware of any information on complaints processes shared or displayed within the hospital? |
|  | 1. Is this information accessible and easy to understand for patients? |
|  | 1. Does your organisation promote learning from complaints with staff? If so, how? |
| B.1 Physical opportunity | 1. How well equipped do you think is the hospital for managing complaints? Are there sufficient resources, systems in place to deal with complaints? |
|  |  |
|  | 1. In your experience, was the complaint(s) responded to or resolved in a timely manner? |
|  | 1. Do healthcare personnel have the required time and access to systems in order to provide the complainant with a regular update and to keep a formal record? |
| B.2 Social opportunity | 1. What is the culture of complaints handling in your hospital? |
|  | 1. Is there blame associated with a complaint? |
|  | 1. Are people who are being complained about, supported during the complaints process? What was the support provided by your manager/organisation? |
|  | 1. What is the reaction of your colleagues? How do your colleagues influence/support you in this process? |
|  | 1. How would you address poor complaints handling from senior members of staff? |
| C.1 Reflective motivation | 1. How confident are you when it comes to complaints management? Do you think complaints handling skills should be part of your role within the hospital? |
|  | 1. Which complaints do you find most difficult to address or resolve? What are the reasons? |
|  | 1. Patients often say that all they want is an apology. We understand that this can be sometimes tricky to do. Do you find it difficult to accept responsibility, in particular for certain types of complaints? |
|  | 1. What type of help or support with would you like in order to better equip you to respond to complaints? What kind of support would make complaint handling more effective? |
|  | 1. Are lessons learned from concerns and complaints and is action taken as a result to improve the quality? Are lessons shared with other colleagues? |
| C.2 Automatic motivation | 1. How does it feel when you receive a complaint? |
|  | 1. How do you contain your emotions? |
|  | 1. Do ever identify with the complainant? (Roughly, how often?) |
|  | 1. What are the possible outcomes or impact of patient complaints on your practice and wellbeing? |
|  | 1. Do you ever identify with other colleagues complained about—do you feel sorry for them? Roughly, how often? |

Supplementary file 3 – Topic guide for patients and carers

| **COM-B Construct** | **Questions** |
| --- | --- |
| A1. Psychological capability | 1. How did you find out what the process is for complaining? |
|  | 1. Were you given any leaflets or information about complaints procedures at the hospital at any point? |
|  | 1. How did you find out how to complain? If you were not given any information, how easy or difficult was it for you to find this information? |
|  | 1. Was the outcome explained to you in plain language and sufficiently? Did you think they were honest and transparent with you?   (For PALS: do people who use the service know how to make a complaint or raise concerns) |
|  | 1. Based on your experience, is the outcome explained appropriately to the complainant in your local hospital? Is there openness and transparency about how complaints and concerns are dealt with? |
|  | 1. Is there any information on complaints shared or displayed within the hospital? Is this information accessible and easy to understand? |
|  | 1. Do you think the staff have the necessary skills to handle effectively a complaint? |
| B1. Physical opportunity | 1. Based on your experience, how easy/accessible is it for you /other people to use the system to look for information or if they have to file a report or write a letter/email to complain or to raise concerns? |
|  | 1. Do you think it is particularly difficult for certain groups of people (and why)? |
|  | 1. In your experience, was the complaint(s) responded to or resolved in a timely manner? |
| B2. Social opportunity | 1. Do you think the healthcare personnel had the necessary time that is required to examine and address your complaint sufficiently? |
|  | 1. Were you given any updates about the progress of your complaint during the period that the process lasted? Was this important/necessary for you? (i.e., to get regular updates regarding the complaint process?) |
|  | 1. Do you think staff have the time/access to systems in order to provide the complainant with a regular update and to keep a formal record? |
|  | 1. Did you get any support when making the complaint? (either from PALS or from any other group) |
|  | 1. Would you have liked to have any/more support or did you not need it? |
|  | 1. Were you encouraged to make a complaint or discouraged? |
|  | 1. Based on your experience, are people treated compassionately and given the help and support they need to make a complaint when required? |
|  | 1. Based on your experience, are people who want to make a complaint supported by advisory groups during the complaints process? |
| C.1 Reflective motivation | 1. What types of responses would you like to see from staff more? |
|  | 1. What changes would you like to see? |
|  | 1. Based on your experience, do you think there are particular groups of healthcare professionals more willing to help than others? (or some more unwilling to handle the complaints/dismiss the complaint/refer you elsewhere?) |
|  | 1. Judging from your experience, what do you think are lessons to be learned from concerns and complaints? Do you think staff in your hospital have learned from previous complaints? Do you have any indication about this? |
|  | 1. Were you asked at any point what your expectation were when you made your complaint? Did they try to respond accordingly? |
|  | 1. Do you think there should be some action to be taken in the hospital to improve the quality of responses? |
|  | 1. Are lessons from complaints shared with others (Q for PALS)? |
|  | 1. Q for PALS: What are the difficulties in handling complaints (inherent conflicts) as part of your role? Do you think you represent all partied equally? If not, what are the problems? |
| C.2 Automatic motivation | 1. Did you feel confident speaking up for yourself and expressing your dissatisfaction with the level of care you received? |
|  | 1. How do you feel when your complaint is not being addressed the way you would have hoped? |
|  | 1. Has this process affected your family? |
|  | 1. If yes, what was/is the impact of the complaints process on you or your family? |
